# Genome-Wide Identification and Characterization of Growth Regulatory Factor Family Genes in *Medicago*

**DOI:** 10.3390/ijms23136905

**Published:** 2022-06-21

**Authors:** Wenxuan Du, Junfeng Yang, Qian Li, Qian Su, Dengxia Yi, Yongzhen Pang

**Affiliations:** 1 Institute of Animal Science, Chinese Academy of Agricultural Sciences, Beijing 100193, China; n053727@163.com; 2 College of Horticulture, Hunan Agricultural University, Changsha 410128, China; yangjf63@sina.com; 3 West Arid Region Grassland Resource and Ecology Key Laboratory, College of Grassland and Environmental Sciences, Xinjiang Agricultural University, Urumqi 830052, China; qianli20210715@163.com; 4 Key Laboratory of Forage and Endemic Crop Biotechnology, Ministry of Education, School of Life Sciences, Inner Mongolia University, Hohhot 010010, China; suqian2021425@163.com

**Keywords:** *Medicago truncatula*, *Medicago sativa*, *GRF* genes, abiotic stresses, expression profiling

## Abstract

Growth Regulatory Factors (GRF) are plant-specific transcription factors that play critical roles in plant growth and development as well as plant tolerance against stress. In this study, a total of 16 *GRF* genes were identified from the genomes of *Medicago truncatula* and *Medicago sativa*. Multiple sequence alignment analysis showed that all these members contain conserved QLQ and WRC domains. Phylogenetic analysis suggested that these GRF proteins could be classified into five clusters. The *GRF* genes showed similar exon–intron organizations and similar architectures in their conserved motifs. Many stress-related *cis*-acting elements were found in their promoter region, and most of them were related to drought and defense response. In addition, analyses on microarray and transcriptome data indicated that these *GRF* genes exhibited distinct expression patterns in various tissues or in response to drought and salt treatments. In particular, qPCR results showed that the expression levels of gene pairs *MtGRF2*–*MsGRF2* and *MtGRF6*–*MsGRF6* were significantly increased under NaCl and mannitol treatments, indicating that they are most likely involved in salt and drought stress tolerance. Collectively, our study is valuable for further investigation on the function of *GRF* genes in *Medicago* and for the exploration of *GRF* genes in the molecular breeding of highly resistant *M. sativa*.

## 1. Introduction

Growth Regulatory Factors (GRFs) are plant-specific transcription factors that are widely distributed in the plant kingdom. Growth Regulatory Factor genes have been reported to play important roles in regulating plant growth and development as well as in plant responsiveness to abiotic stress [1]. Growth Regulatory Factors are a small family of transcription factors, and they contain two signature and conserved functional domains at N-terminal regions, namely the QLQ domain (Gln, Leu, Gln, IPR014978) and the WRC domain (Trp, Arg, Cys, IPR014977) [2,3]. The QLQ domain can interact with GRF Interaction Factor (GIF) to exert their functions [4]. The WRC domain functions in DNA binding and transcription factors targeting the nucleus, which consist of a C3H motif for DNA binding and a nuclear localization signal (NLS) [5]. The C-terminal region of GRF is variable when compared with the conservative amino acid residues in the N-terminal region, and this region has the transactivation activity [4,5,6,7]. In addition, several other motifs, such as TQL (Thr, Gln, Leu) and FFD (Phe, Phe, Asp), are usually present in the C-terminal region of GRFs, although they are not highly conservative [8].

*GRF* genes participate in the early growth and development of plants, and they play an important regulatory role in the formation of plant tissues or organs, such as leaf development, stem elongation and root growth [9,10]. These transcription factors directly affect the morphological establishment of the plant, which in turn affects plant yield. *OsGRF1* from deepwater rice (*Oryza sativa*) was the first member identified in plants, which encodes a protein that could induce stem elongation by regulating gibberellins biosynthesis [2]. It was also reported that GRF could regulate the shape and size of leaves by regulating cell proliferation [11,12]. In the model plant *Arabidopsis thaliana*, the overexpression of *AtGRF1*, *AtGRF2* and *AtGRF5* resulted in larger leaves than the wild-type plant but much smaller leaves in the *grf* mutants than in the wide type, including mutants of *grf3-1*, *grf5-1*, *grf1-1*/*grf2*, *grf2*/*grf3* and *grf1*/*2*/*3* [3,11,12,13]. *GRF* genes may act by regulating cell proliferation through the suppression of *KNOX* gene expression [14], which inhibits GA biosynthesis in the S-adenosyl methionine (SAM) cycle by down-regulating the key biosynthetic gene GA20 oxidase [15] or by controlling the level of GA2 oxidase 1 that degrades GA [16]. Recently, many studies have reported the involvement of *GRF* genes in the regulation of flower development [17].

Most *GRF* genes are the target genes of microRNA396 (miR396). Evidence showed that miR396 can directly inhibit *GRF* expression through post-transcriptional regulation [17]. *AtGRF1-4* and *AtGRF7-9* are the target genes of miR396 in *Arabidopsis* [1]. The expression of miR396 is induced by various types of abiotic stresses such as high salinity, low temperature, drought stress, and UV-B as well as TCP transcription factors (TCP4) [18]. Interestingly, the overexpression of miR396 resulted in a decreased expression of *AtGRF6*, although it was not a target of miR396 [19]. Furthermore, GIF interacts with GRFs, floral identity factors [20], and chromatin remodeling complexes [21] to regulate reproductive competence and organogenesis [14]. Therefore, the transcript level of GRF is regulated by the miRNA–GRF–GIF cascade.

The expression of GRFs responds to certain abiotic stresses. In *Arabidopsis*, the expression level of *AtGRF7* is inhibited under high salt and drought conditions to activate osmotic stress response genes [22]. The functional classification of downstream genes of *AtGRF1* and *AtGRF3* indicates that most of their target genes are involved in stress defense responses [4]. This study indicated that GRF is also involved in resistance to stress adversity. Plant morphogenesis is influenced by plant growth, development, regulatory capacity, and environment [23,24]; thus, the *GRF* gene family can regulate both the growth and development of the aboveground part to improve yield as well as the development of roots to increase stress resistance. With the release of plant reference genomes, members of the GRF gene family have been identified in several plant species, such as 9 members in *Arabidopsis thaliana* [3], 12 in rice (*Oryza sativa*) [5], 18 in soybean (*Glycine max*) [25], 14 in maize (*Zea mays* L.) and 12 in tomato (*Lycopersicon esculentum*) [26]. Although the functions of some GRF proteins in model plant species have been identified, the information on the members, characteristics, and functions of the *GRF* gene family in legume *Medicago* is still unknown.

*Medicago sativa* is a perennial legume that is widely used as an important forage crop with high yield and quality. *M. sativa* is rich in protein, minerals, vitamins and other nutrients, which is the optimal forage for improving the quality of livestock products [27,28]. However, due to the genetic complexity of tetraploid *M. sativa* and its intolerance to environmental stress, its yield and quality improvement have been restricted [28]. *M. truncatula* is a close relative of *M. sativa* that has been developed as a model legume species. These genetic advantages and genomic resources of *M. truncatula* make the research on *M. sativa* convenient. In this study, the *GRF* family genes were identified and analyzed in two *Medicago* species. Multiple sequence alignment, phylogenetic relationship, gene structure, protein motifs and *cis*-acting elements were systematically analyzed. In particular, the expression profiles of *GRFs* in response to salt and drought stress were analyzed in *M. truncatula* and *M. sativa*. Our results also identified potential new *GRF* genes for genetic modification in *M. sativa*.

## 2. Results

### 2.1. Identification of GRF Genes in the M. sativa and M. truncatula Genome

A total of eight candidate *GRF* genes were obtained from the *M. truncatula* genome and eight were obtained from *M. sativa*. Characteristics of *GRF* genes, including TIGR locus, chromosome location, homologous gene, isoelectric point (pI), molecular weight (MW), and putative subcellular localization, which are listed in Table 1. As a result, the corresponding predicted precursor proteins of *MtGRF*/*MsGRF* varied from 325 to 654 aa and 157 to 516 aa, respectively. We also found the pI value ranges of MtGRF and MsGRF were 7.07–9.03 and 6.28–10.15 kDa, respectively. In addition, the corresponding MW of MtGRF and MsGRF ranges were 36.40–70.74 and 17.55–56.20 kDa, respectively. Moreover, the corresponding homologous *GRF* genes of *M. truncatula* and *M. sativa* were identified based on sequence alignment. Subcellular location analysis showed that most of the predicted GRF proteins from *M. truncatula* and *M. sativa* were located in the nucleus or extracellularly (Table 1).

### 2.2. Multiple Sequence Alignment, Phylogenetic Analysis and Classification of GRF Genes in Medicago

**Figure 1:**
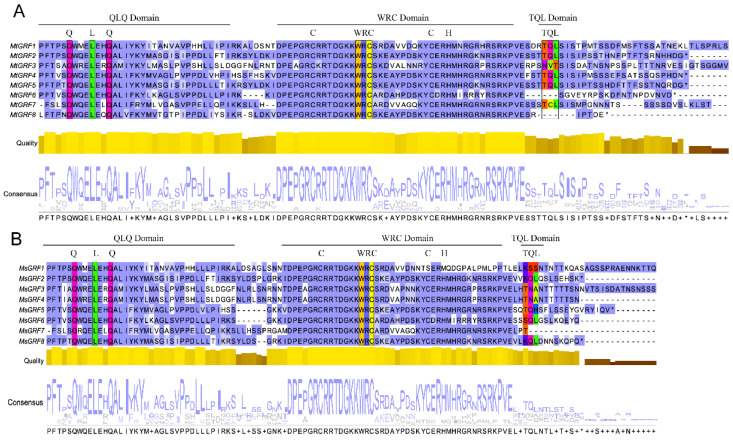
Multiple sequence alignment of MtGRFs (**A**) and MsGRFs (**B**). The result only showed partial sequences containing the QLQ, WRC and TQL domains. The alignment was constructed by using MEGA-X and visualized by using Jalview. Residues with more than 50% similarity were shaded. Conserved regions (QLQ, WRC, and TQL) were indicated at the top.

In order to better understand the characteristics of the GRF protein sequence, the most conservative region covering QLQ and WRC domains was analyzed (Figure 1A,B). It was shown that all 16 GRF proteins shared the same QLQ and WRC amino acids, and the sequences were highly conserved among these two domains (Figure 1). In contrast, the TQL domain was only present in the C-terminal of some GRF members that were similar to those GRFs members from *Arabidopsis* [3].

**Figure 2:**
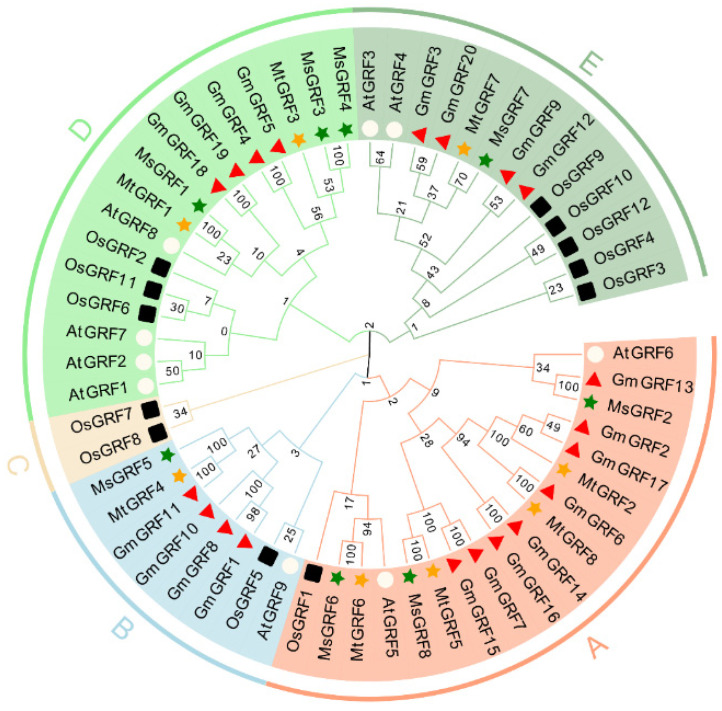
Phylogenetic analysis of GRF families across *Medicago*, *Arabidopsis*, *G. max* and *O. sativa*. Full-length protein sequences of GRFs were constructed using MEGA-X based on the Neighbor-Joining (NJ) method; bootstrap was 1000 replicates. Subfamilies were highlighted with different colors. The green solid pentagrams, orange solid pentagrams, hollow circles, red triangle and black square represent GRF proteins from *M. truncatula* (Mt), *M. sativa* (Ms), *A. thaliana* (At), *G. max* (Gm) and *O. sativa* (Os), respectively.

Sequence-based phylogenetic analysis among *M. truncatula*, *M. sativa*, *G. max*, *O. sativa* and *Arabidopsis* showed that these proteins were grouped into five distinct clusters (A–E, Figure 2). The largest cluster was group A with 18 members from all five species, and the smallest cluster was group C with two members (*OsGRF7* and *OsGRF8* from rice) (Figure 2), and they may be unique for monocot plants such as rice. Cluster A and D contained the most GRF members from *Medicago*, with Cluster A containing three MsGRFs and two MtGRFs, and cluster D containing three MsGRFs and four MtGRFs. Cluster C and E contained one member from *M. truncatula* and *M. sativa*, respectively (Figure 2).

### 2.3. Analyses of Conserved Motif and Gene Structure

**Figure 3:**
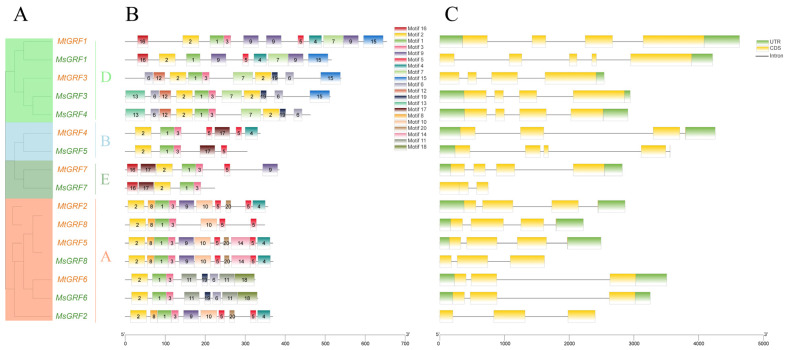
Phylogenetic relationships, motifs and gene structure of *GRF* genes from *M. truncatula* and *M. sativa* (**A**–**C**). The groups and its color in the phylogenetic tree were the same as in Figure 2. The motifs were indicated in different colored boxes with different numbers, and the sequence information for each motif was provided in Additional Figure 1. Green boxes indicate 5′- and 3′-untranslated regions; orange boxes indicate exons; black lines indicate introns.

To identify the conservative structure of Ms/Mt GRF proteins, 20 motifs were analyzed through the MEME program (Supplementary Figure S1), and their positions were illustrated on each gene (Figure 3B). Most of them had similar motif positions and types. GRF members with fewer motif numbers and types were found for sub-clusters A, B and E, whereas sub-cluster D contained more numbers and types. All GRF members contain motifs 1 and 2, and the homologous gene pairs (*MtGRF2*/*MsGRF2*, *MtGRF5*/*MsGRF8*, and *MtGRF6*/*MsGRF6*) had identical motif structures; it is suggested that they may share the same roles in *Medicago*.

Analysis of the *GRF* genes structure showed that they had one to four introns and two to four exons. In particular, *MsGRF7* had only one intron (Figure 3C). Nevertheless, some of the *MsGRF* genes lacked the 5′-UTR or 3′-UTR, indicating that their sequences are incomplete, which may be due to the genome assembly.

### 2.4. Analysis of Chromosome Location and Collinearity of GRF Genes

**Figure 4:**
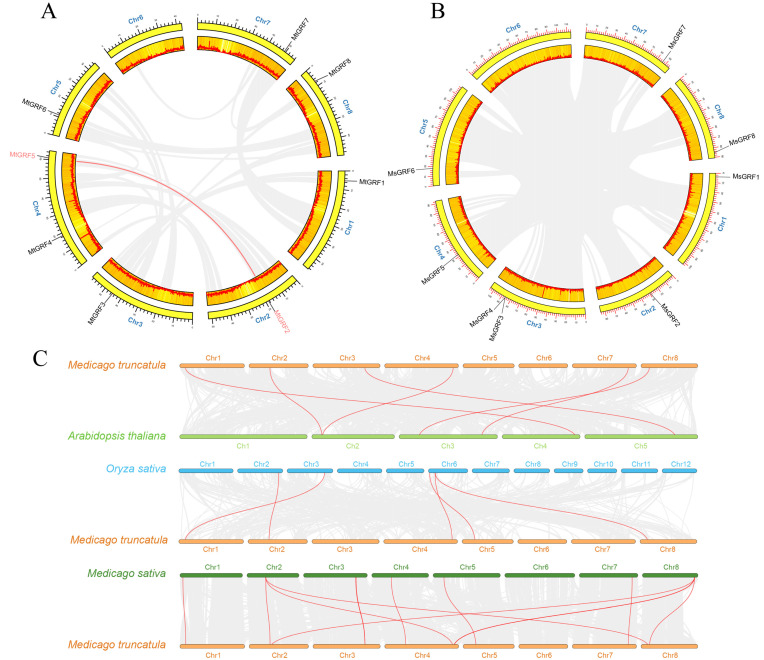
Chromosome distributions of GRFs in *M. truncatula* and *M. sativa*. The chromosomal location and interchromosomal relationship of *M. truncatula* (**A**) and *M. sativa* (**B**). The segmentally duplicated genes are connected by red curves. (**C**) Synteny analysis of *GRF* genes between *A. thaliana* and *M. truncatula*, *O. sativa* and *M. truncatula*, *M. sativa* and *M. truncatula*. Gray lines in the background indicate the collinear blocks between *M. truncatula*, and *A. thaliana*/*O. sativa*/*M. sativa*, and the red lines highlight the syntenic *GRF* gene pairs.

The distribution of *GRF* genes was not even in either *M. truncatula* or *M. sativa*, and they were distributed on seven chromosomes, except for chromosome 6. Four homologous gene pairs (*Mt*/*MsGRF1*, *Mt*/*MsGRF2*, *Mt*/*MsGRF6*, *Mt*/*MsGRF7*) were distributed in four chromosomes (Chr1, 2, 6, 7) of *M. truncatula* and *M. sativa*, respectively, and one *GRF* gene was distributed in each chromosome (Figure 4A,B). In *M. truncatula*, the remaining *GRF* members *MtGRF3*, *4*, *5* and *8* are distributed in chromosomes 3, 4, 4, and 8, respectively. In *M. sativa*, the remaining GRF members *MsGRF3*, *4*, *5* are distributed in chromosomes Chr3, 3, 4, and 8, respectively (Figure 4A,B).

To further investigate the evolutionary mechanism of the *GRF* gene family, both tandem and segmental duplication events were analyzed. Only one *MtGRF* gene pair (*MtGRF2*/*MtGRF5*) could be identified as segmental duplication events, but neither segmental duplication nor tandem duplication was identified in *M. sativa* (Figure 4A,B).

Comparative syntenic maps of *A. thaliana*, *O. sativa* and *M. sativa* associated with *M. truncatula* were constructed to illustrate the evolution relationship of the *GRF* gene family (Figure 4C). Notably, 6, 5 and 11 orthologous pairs were found between *A. thaliana* and *O. sativa*, *M. sativa* and *M. truncatula*, *M. truncatula* and *M. sativa*, respectively (Supplementary File S1). Four genes in *M. truncatula* (*MtGRF1*, *2*, *5* and *8*) showed a collinear relationship with those in *A. thaliana*, *O. sativa* and *M. sativa*, respectively (Figure 4C). These genes may play an irreplaceable role in the evolution of the *GRF* family.

To better understand the evolutionary selection pressure during the formation of the *GRF* gene family, the Ka/Ks values of *GRF* gene pairs were analyzed for both *M. truncatula* and *M. sativa* (Supplementary File S1). The Ka/Ks values of the *M. truncatula* and *M. sativa* orthologous gene pairs were all less than 1. Taken together, these results indicated that the *GRF* genes of *M. truncatula* and *M. sativa* may have undergone strong purification selection pressure during evolution.

### 2.5. Analysis of cis-Acting Element of GRF Genes

**Figure 5:**
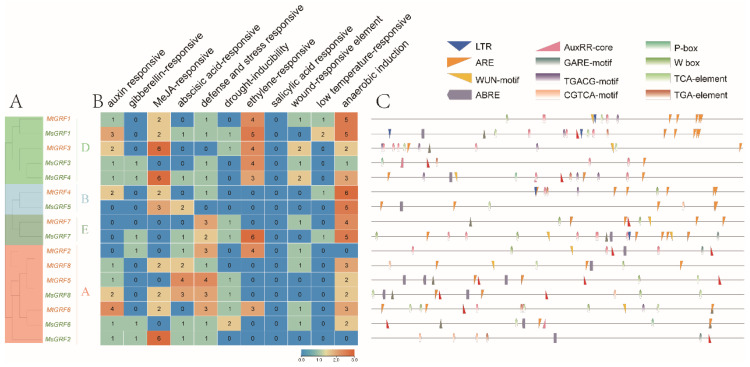
Putative *cis*-elements and transcription factor binding sites in the promoter regions of the *GRF* genes from *M. truncatula* and *M. sativa*. (**A**) The groups and color are indicated as in Figure 2. (**B**) The color and number of the grid indicated numbers of different *cis*-acting elements in these *GRF* genes. (**C**) The colored block represented different types of *cis*-acting elements and their locations in each *GRF* gene.

The *cis*-acting elements are important for the binding of transcription factors, which control the expression of their downstream target genes. The promoter sequence of 2000 bp for the eight *MtGRF* and eight *MsGRF* genes were analyzed. Several different types of *cis*-acting elements were identified, including: auxin responsive (AuxRE-core), gibberellin-responsive (GARE-motif, P-box, TATC-box), MeJA-responsive (TGACG-motif, CGTCA-motif), abscisic acid-responsive (ABRE), defense and stress responsiveness (TC-rich repeats, W-box), MYB binding site involved in drought-inducibility (MBS), ethylene-responsive (ERE), salicylic acid responsiveness (TCA-element), wound responses (WUN motif), low-temperature responsive (LTR), and anaerobic induction (ARE) (Figure 5 and Supplementary File S2).

With an emphasis on defense and stress-related *cis*-acting elements, we found that the promoter of *MtGRF6* and *MsGRF7* had at least two W-box repeat elements, and *MsGRF6* contained two MBS repeat elements (Figure 5B,C). Moreover, most *Mt*/*MsGRFs* contained many *cis*-elements related with anaerobic induction elements (Figure 5B,C), which may play a regulatory role under root hypoxic conditions. Notably, *GRF* genes with a high number of ethylene-responsive elements were clustered in the D group. These genes may play a key role in promoting plant development and maturation.

### 2.6. Expression Profiles of GRF Genes in Different Tissues

**Figure 6:**
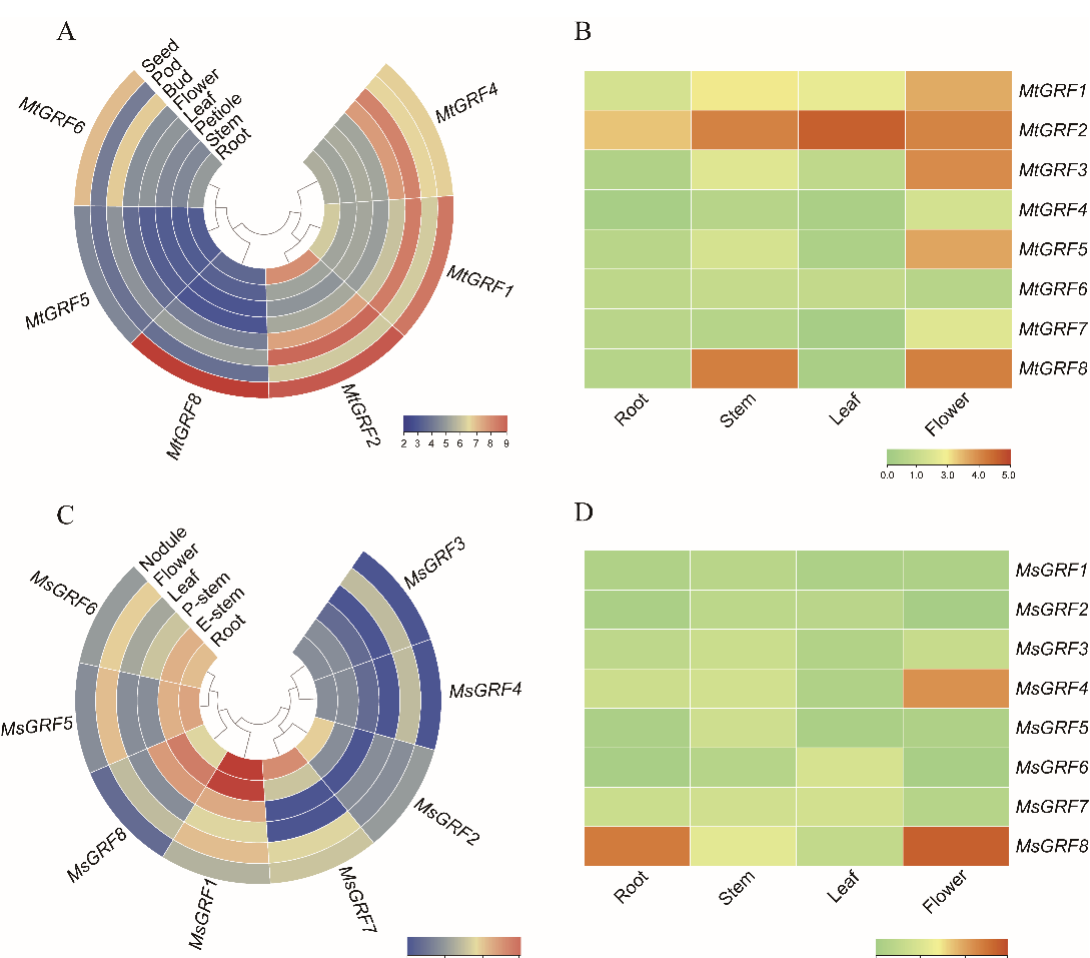
Expression profiles of *GRF* genes in various tissues. (**A**,**C**): Expression profiles of *MtGRF* and *MsGRF* genes in different tissues retrieved from genechip dataset and transcriptome, respectively. (**B**,**D**): Expression level of *MtGRF* and *MsGRF* genes in various tissues verified by qPCR. The relative expression levels are log2-transformed and visualized for heatmap. Red represents relatively high expression and blue (**A**,**C**) or green (**B**,**D**) represents relatively low expression.

We investigated the expression profiles of *GRFs* in various tissues of *M. truncatula* with the genechip dataset, including roots, stems, leaves, flowers, pods, petioles, seeds and buds (Figure 6A). Remarkably, three genes (*MtGRF4*, *1*, *2*) showed a relatively high expression level in these tissues, whereas *MtGRF5* and *MtGRF6* were expressed at a relatively low level in different tissues (Figure 6A). Their expression levels in four tissues (roots, stems, leaves and flowers) were further verified by qPCR (Figure 6B, Supplementary File S5). Notably, the expression level of *MtGRF2* was high than that of other genes, whereas that of *MtGRF6* was relatively low in all four tissue. This was consistent with the results as shown in Figure 6A. In addition, *MtGRF1*, *3*, *4*, *5*, and *7* were highly expressed in flowers, and *MtGRF8* was more highly expressed in both stems and flowers than in other tissues (Figure 6B).

Six tissues from *M. sativa* were analyzed based on transcriptome data, including roots, elongated stems, pre-elongated-stems, leaves, flowers and nodules. Among them, *MsGRF1* showed relatively high expression level in various tissues, especially in roots and elongated stems. Three genes (*MsGRF2*, *3*, *4*) were expressed at a relatively low level in all tissues. *MsGRF7* gene was expressed at a higher level than in other tissues. *MsGRF5*, *6*, and *8* were expressed at a relatively higher in elongated stems and flowers than in other tissues (Figure 6C). Using qPCR analysis, we found that *MsGRF4* showed the highest expression level in flowers (Figure 6D), which was consistent with that in Figure 6C. Analysis revealed that the expression level of *MsGRF8* gene was much higher in roots and flowers than in stems and leaves, which was even higher than those of all other *MsGRF* genes (Figure 6D).

### 2.7. Expression Profiles of MtGRF Genes under Stress Treatments

Expression profiles of *GRF* genes from *M. truncatula* were initially analyzed based on the data retrieved from the MtGEA web server, including samples from roots and shoots under drought treatment, and roots under vitro culture salinity and under hydroponic salinity (Supplementary Figure S1). One probe set was selected as representative for each *MtGRF* gene, and six out of eight *MtGRF* genes have their corresponding probe set: *MtGRF1*, *2*, *4*, *5*, *6*, *8* (Supplementary File S4).

**Figure 7:**
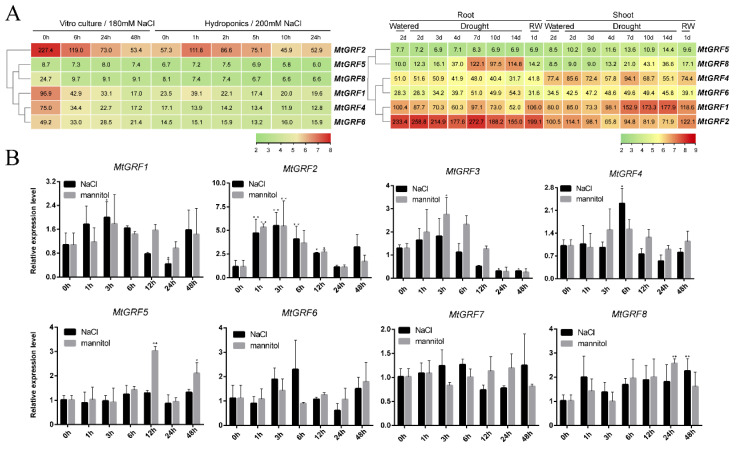
Expression profiles of *MtGRF* genes under NaCl and mannitol treatment. (**A**) Left, *MtGRF* genes expression level under drought treatment in roots and in shoots at different treatment times. Right, *MtGRF* genes expression level under vitro culture and hydroponics culture at different treatment times. (**B**) qPCR analysis on the expression of *MtGRF* genes treated with 300 mM NaCl and 15% mannitol at 0 h, 1 h, 3 h, 6 h, 12 h, 24 h, and 48 h. Data are the average of three independent biological samples ± SE, and vertical bars indicate standard deviation. ** indicates *p* < 0.01, and * for *p* < 0.05.

Under drought conditions, the expression levels of *MtGRF1*, *MtGRF2*, *MtGRF6* and *MtGRF8* were highly induced in both roots and shoots (Figure 7A, left panel). Specifically, the expression level of several genes was significantly increased under drought, but it decreased after re-watering. These include *MtGRF8* and *MtGRF6* in roots and *MtGRF5*, *MtGRF8*, *MtGRF1* and *MtGRF6* in shoots. In contrast to drought treatment, the expression level of all six *MtGRF* genes decreased under NaCl treatment (Figure 7A, right panel).

To further understand the potential roles of *MtGRF* genes under abiotic stress, seedlings were treated with 300 mM NaCl and 15% mannitol, respectively. The expression levels of *MtGRF* genes were analyzed at 0 h, 1 h, 3 h, 6 h, 12 h, 24 h, and 48 h for two treatments by qPCR analysis (Figure 7B). It was shown that the expression level of almost all *MtGRFs* changed differently from the control in both treatments (Figure 7B). The detailed results were also displayed in Supplementary File S5. Notably, *MtGRF2* was highly induced in response to both NaCl and mannitol treatment at 1 h and 3 h (Figure 7B). All genes except *MtGRF7* were significantly up-regulated in response to at least one treatment at different time intervals. For example, *MtGRF4* and *MtGRF6* were up-regulated at 6 h for NaCl treatment, *MtGRF3* at 3 h, 6 h, *MtGRF5* at 12 h, 24 h, and *MtGRF8* at 48 h, under mannitol treatment (Figure 7B).

### 2.8. Expression Profiles of MsGRF Genes under Stress Treatments

**Figure 8:**
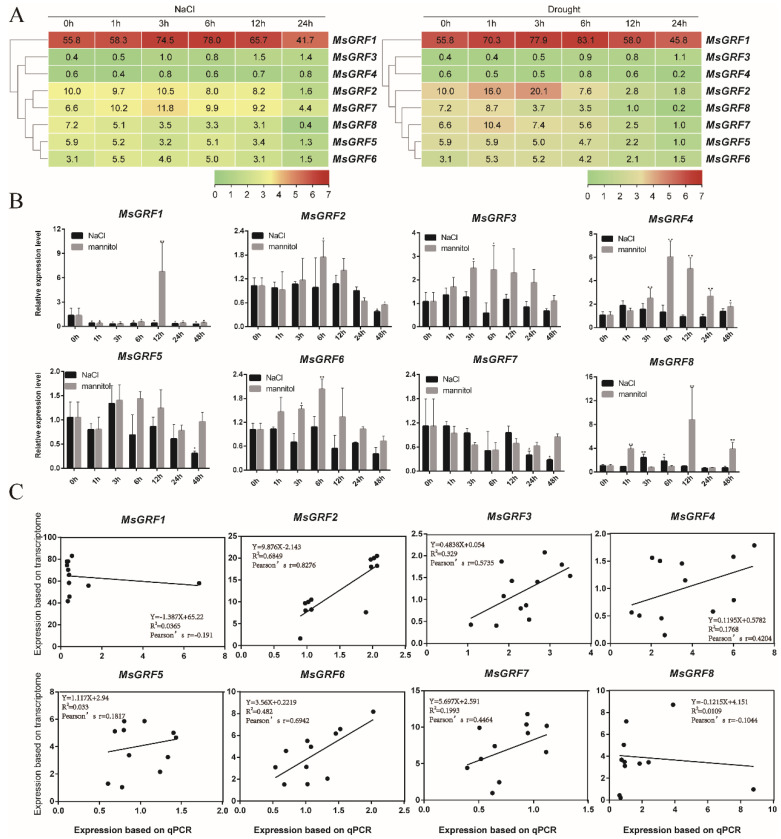
Expression profiles of *MsGRF* genes under NaCl and mannitol stress. (**A**) *MsGRF* genes expression in roots under NaCl treatment at different time points; in root under drought treatment at different time points. (**B**) qPCR analysis on the expression level of *MsGRF* genes treated with 300 mM NaCl and 15% mannitol at 0 h, 1 h, 3 h, 6 h, 12 h, 24 h, and 48 h. Data are the average of three independent biological samples ± SE, and vertical bars indicate standard deviation. ** indicates *p* < 0.01, and * for *p* < 0.05. (**C**) Correlation analysis of qPCR and transcriptome data for *MsGRF* genes. Pearson’s r indicates the Pearson correlation coefficient.

The expression levels of *MsGRF* genes were analyzed under NaCl and drought treatment with transcriptome data. It was found that most genes were induced at different levels (Figure 8A). Under both treatments, *MsGRF1* maintained a relatively higher level than all the other genes (Figure 8A). The expression levels of *MsGRF3*, *MsGRF7*, and *MsGRF6* were slightly increased at 1 h under NaCl treatment (Figure 8A, left), and those of *MsGRF2*, *MsGRF8*, *MsGRF7*, and *MsGRF6* were slightly increased at 1 h under drought treatment (Figure 8A, right).

qPCRs were performed to verify the expression of all *MsGRF* genes at the same time intervals with both NaCl and mannitol (as drought) treatment (Figure 8B). Our results showed that all genes except *MsGRF5* and *MsGRF7* were up-regulated in response to at least one treatment. All *MsGRF* genes were significantly more sensitive to mannitol treatment than to NaCl treatment (Figure 8B). In particular, *MsGRF1*, *MsGRF3*, *MsGRF4*, and *MsGRF8* responded remarkably at 12 h under mannitol treatment (Figure 8B). Correlation analysis between qPCR data and transcriptome data showed that they were positively correlated for six genes *MsGRF2-7* (Figure 8B). In particular, the co-efficiency values were much higher for *MsGRF2* and *MsGRF6* than for the other four genes (*MsGRF3*, *4*, *5*, *7*, Figure 8C).

## 3. Discussion

Growth Regulatory Factors are plant-specific transcription factors that regulate early plant morphogenesis and root development, and they play a critical role in the genetic improvement of crops on yield and resistance [29]. In general, the number of GRF members in plants ranged from 8 to 20 [30]. In the current study, eight *MtGRF* and eight *MsGRF* genes were identified in *M. truncatula* and *M. sativa*, respectively.

Multiple sequence alignment confirmed that all GRF members from *M. truncatula* and *M. sativa* contained the QLQ domains and WRC motifs. All of them were present at the N-terminus (Figure 1). This observation was consistent with those from model plants such as rice [2], *Arabidopsis* [3], and soybean [25]. We found that two structural domains at the N-terminus determine the basic architecture of the *GRF* family in *Medicago*. Moreover, we performed gene annotation based on the presence of these motifs. Motif1 was associated with WRC domains, whereas motif2 was annotated as an ATP-binding domain and associated with the QLQ domain. Notably, in *M. truncatula*, the TQL domain at the C-terminus was present only in MtGRF1, 2, 4, 5 and 7, whereas *M. sativa* did not contain a TQL domain. Thus, we speculated that the functional diversity of GRF proteins is based on the diversity of the C-terminal domain. Phylogenetic tree analysis revealed that the GRF members of *Medicago* clusters were identical to those of *Arabidopsis* [31].

Duplication and divergence play critical roles in the expansion and evolution of gene families [29,32]. We found only one segmental duplication among eight *MtGRFs* (Figure 4), while no duplication events were found in *M. sativa*. This indicates that *GRF* genes were conservative during the evolution of *Medicago*. The combination of phylogenetic and collinearity analyses based on gene expression is valuable for understanding the function of *GRF* genes in specific physiological processes. For example, by analyzing the genechip data (Figure 6), we found that the transcript levels of *MtGRFs* were higher in seeds and buds than in the other tissues examined, whereas previous studies have shown that in other plant species, the transcript levels of *GRFs* are higher in young leaves. These findings suggest that *GRFs* in *Medicago* may function primarily in regulating seeds and buds development. It was reported that *AtGRF7*, a gene homologous to *MtGRF5*, can bind the DREB2A promoter and repress its expression under non-stress conditions. It should be noted, however, that abiotic stress suppresses *AtGRF7* expression, thereby activating osmotic stress-responsive genes [22]. In our study, *MtGRF5* contains more ABRE elements (ABA responsive) and can possibly respond effectively to osmotic stress. It is possible therefore that *MtGRF2* and *MtGRF5* may share a similar function in the regulation of osmotic stress in *Medicago* as *AtGRF7*.

Since salinity and drought soils are the most prevalent and severe abiotic stresses affecting plant growth [33], it is extremely important to improve salt and drought tolerance in plants such as *M. sativa*. Analyses on the expressions level of several *GRF* genes were either highly induced or drastically changed under NaCl and mannitol treatments (Figure 7 and Figure 8). Meanwhile, the expression of several *GRF* genes were verified to be up-regulated under two treatments in *M. truncatula* and *M. sativa* (Figure 7 and Figure 8). *MtGRF2* and *MtGRF8* showed high expression levels under drought stress (Figure 6B), which was consistent with their expression level in roots as analyzed by microarray data. All these data indicates that they may play an important role in resistance against abiotic stress in roots. Correspondingly, two gene pairs (*MtGRF2*/*MsGRF2* and *MtGRF6*/*MsGRF6*) were significantly up-regulated under both treatments, and their expression patterns were the same in *M. truncatula* and *M. sativa.* By identifying the *cis*-acting elements’ bound specific transcription factors, it is possible to reveal the transcriptional regulatory mechanism and gene expression patterns during plant environmental adaptation. Since *MtGRF6* had at least two W-box repeat elements, and since *MsGRF6* contained two MBS repeat elements (Figure 5C), they may play key roles in increasing the stress resistance of *M. truncatula* and *M. sativa.*

Previous studies have confirmed that GRF functions by regulating the complex process of plant growth and responses to environmental stress [22]. For example, *OsGRF1* may regulate gibberellic acid (GA)-induced stem elongation and transcriptional activity [2]. Meanwhile, in plants overexpressing *AtGRF5*, the exit of the cell proliferation phase is delayed in early leaf development and chloroplasts divide extensively, while the onset of the cell expansion phase is delayed. Cytokinins are thought to increase the number of chloroplasts in the cell and act synergistically with *AtGRF5* to increase photosynthesis rates, thereby increasing leaf size, plant productivity, and leaf longevity [34]. Notably, the homologous genes (*MtGRF6*/*MsGRF6*) are more closely related to *AtGRF5* and *OsGRF1*, and this gene pair exhibited high expression under NaCl and mannitol stress, indicating that these two genes are possibly involved in early leaf development and transcriptional activity. *OsGRF6* positively regulates auxin synthesis, promotes inflorescence development, and increases spike number [34]. The closely related genes in *Medicago* were *MtGRF1* and *MsGRF1*, and they may also play a similar role in plant development. In *A. thaliana*, plants overexpressing *AtGRF9* significantly inhibited the growth of leaves [35]. Its closely related gene was *MsGRF5* in *M. sativa*, which was down-regulated under NaCl treatment, suggesting that this gene may be involved in stress response and the promotion of plant growth. These results suggested the potential roles of *GRF* genes in *M. truncatula* and *M. sativa* under abiotic stresses resistance. Not all these homologous genes in *M. truncatula* and *M. sativa* exhibit the same expression pattern under different stress treatments. It is possible that these genes have been subjected to varying degrees during species evolution.

## 4. Conclusions

This study analyzed the *GRF* genes on a genome-wide scale in *M. sativa* and *M. truncatula*. A total of eight *MsGRFs* and eight *MtGRFs* were identified, respectively, in *M. sativa* and *M. truncatula*. These genes show highly similarity in amino acid sequence, motif compositions and conservative gene structure. In addition, phylogenetic analysis and collinearity analysis on GRF in different species revealed their evolutionary patterns and predicted their functions in complex environments. Moreover, the expression profile of *GRF* genes in different tissues and two stress treatments were analyzed and further verified by qPCR. It was found that most genes were highly expressed, especially *MtGRF2*–*MsGRF2* and *MtGRF6*–*MsGRF6*. These gene pairs showed the same expression pattern in *M. truncatula* and *M. sativa*, and they may play an important role in responses to stresses. This study compares the GRF family genes of *M. truncatula* and *M. sativa,* which provide new clues for understanding their evolutionary relationship and functions under abiotic stresses.

## 5. Materials and Methods

### 5.1. Identification of GRF Genes in the Medicago Genome

The genomic data of *M. truncatula* and *M. sativa* were downloaded from the websites https://figshare.com/articles/dataset/Medicago_sativa_genome_and_annotation_files/12623960 (accessed on 1 September 2021) and http://www.medicagogenome.org/ (accessed on 1 January 2020), respectively. To identify all putative GRF transcription factor proteins in each genome assembly, the conserved domains of the GRF protein (PF08879 for WRC domain and PF08880 for QLQ domain) [5] of Hidden Markov Model (HMM) profiles were downloaded from the Pfam protein family database (https://pfam.xfam.org/) (accessed on 11 September 2020). Subsequently, the GRF protein sequences from *M. truncatula* and *M. sativa* were deduced with HMM as a query (*p* < 1 × 10^−5^). Moreover, the *GRF* gene sequences of *Arabidopsis* were downloaded from the TAIR website (https://www.arabidopsis.org/) (accessed on 11 September 2020). In order to further screen the *GRF* genes, output putative GRF protein sequences were submitted to InterProScan (https://www.ebi.ac.uk/interpro/search/sequence-search) (accessed on 12 September 2020), CDD (https://www.ncbi.nlm.nih.gov/Structure/bwrpsb/bwrpsb.cgi) (accessed on 12 September 2020), Pfam (https://pfam.xfam.org/) (accessed on 12 September 2020), and SMART (http://smart.embl-heidelberg.de/) (accessed on 13 September 2020). Finally, 8 *MsGRF* and 8 *MtGRF* genes were identified and assigned based on their locations on chromosome. Correspondingly, ExPASy (https://web.expasy.org/compute_pi/) (accessed on 15 September 2020) was used to determine the isoelectric point (pI) and molecular weight (MW) of GRF proteins. Subcellular localization of GRF proteins were predicted by using the Softberry Home Page (http://linux1.softberry.com/berry.phtm) (accessed on 16 September 2020).

### 5.2. Analyses on Sequence and Structures of the Medicago GRF Genes

Conserved motifs were identified by selecting motifs from the MEME program (http://meme-suite.org/tools/meme) (accessed on 25 September 2020) with the motif number of GRF set as 20 and the width range of 10 to 200 amino acids (aa). Subsequently, sequence alignment was carried out by using jalview (http://www.jalview.org/Web_Installers/install.htm) (accessed on 25 September 2020). The visualization of exon–intron positions and conserved motifs were executed through the TBtools software [36].

### 5.3. Phylogenetic Analysis and Classification of GRF Genes

According to the amino acid sequences of GRF from *M. truncatula*, *M. sativa*, *A. thaliana*, *O. sativa* and *G. max*, the phylogenetic relationship of GRF proteins among these species was analyzed. We constructed a phylogenetic tree based on the complete GRF protein sequences using the Neighbor-Joining method as implemented in the MEGA-X software with a bootstrap value of 1000 replicates [37]. Meanwhile, clustering of the subfamily of GRFs in *Medicago* was based on that of *Arabidopsis*. The online software EvolView (https://evolgenius.info/evolview-v2/) (accessed on 29 September 2020) was used to modify the phylogenetic tree.

### 5.4. Analyses of Chromosome Location and Collinearity of GRF Genes

The chromosome locations of the *GRF* genes were determined using the NCBI website and mapped with the TBtools software. Multiple collinear Scan toolkit (Mcscanx) was used to analyze the gene duplication events with default parameters [38]. The intraspecific synteny relationship (*M. truncatula* and *M. sativa*) and interspecific synteny relationships (*M. truncatula*, *M. sativa*, *Arabidopsis* and *O. sativa*) were analyzed, and they were further mapped to the chromosomes of *M. truncatula* and *M. sativa*, respectively [39]. The simple Ka/Ks calculator software was used to calculate non-synchronous (Ka) and synchronous (Ks) values of *GRF* gene pairs [38].

### 5.5. Analyses of cis-Acting Elements and Location of GRF Genes in Medicago

The promoter sequences (2 kb upstream of the translation start site) of the *GRF* genes were identified by using the TBtools software, and the *cis*-elements in the promoters regions were predicted with the online program PlantCARE (http://bioinformatics.psb.ugent.be/webtools/plantcare/html/) (accessed on 2 October 2020) [40]. TBtools was used to visualize the *cis*-acting elements of all *GRF* genes of *Medicago*.

### 5.6. Analysis of Expression Level of GRF Genes

Genechip data from roots and shoots and those under drought and salt stress conditions for *MtGRF* genes were downloaded from the *M. truncatula* Gene Expression Atlas (https://Mtgea.noble.org/v3/) (accessed on 2 October 2020), and different tissues without stress were also covered. Amazing HeatMap software was used to generate the heatmap [36]. The original transcriptome data from *M. sativa* under NaCl and mannitol treatments at 0, 1, 3, 6, 12, 24 h (SRR7160314-15, 22–23, 25–49, 51–52, 56–57) were downloaded (https://www.ncbi.nlm.nih.gov/sra/) (accessed on 2 October 2020). The data were then converted into fastq files using an SRA-Toolkit v2.9 [40]. Raw reads were trimmed using the Trimmomatic-0.39 [41]. Gene expression level was determined by mapping cleaned reads to the corresponding *M. sativa* reference genomes using the StringTie v2.1.3 package [42].

### 5.7. Plant Materials and Treatments

The *M. truncatula* (cv. Jemalong A17) and *M. sativa* (cv. Zhongmu No. 1) plants used in this study were stored at the Institute of Animal Sciences of Chinese Academy of Agricultural Sciences. Stems, leaves, flowers, and roots (20-day old pods) of mature *M. truncatula* and *M. sativa* plants were collected separately for RNA extraction and qPCR analysis. To investigate the expression pattern of *GRF* genes in response to NaCl and mannitol stress, seeds were germinated and transferred into the MS liquid medium (MS basal salts supplemented with 30 g/L sucrose); then, they were kept in a growth chamber at 25 °C under a photoperiod of 16/8 light/dark regime (80 μmol photons m^−2^ s^−1^) and 80–90% humidity. When the third leaf was fully expanded, 300 mM NaCl and 15% mannitol [39,43] were, respectively, added into the MS liquid medium, and the whole plant was collected at 0 h, 1 h, 3 h, 6 h, 12 h, 24 h and 48 h for each treatment. The samples were frozen in liquid nitrogen and stored at −80 °C for subsequent analysis.

### 5.8. Analysis of Gene Expression by qPCR

Total RNAs were extracted by using an Eastep^®^ Super total RNA Extraction kit (Promega, Shanghai, China) according to the manufacturer’s instructions. First-strand cDNA synthesis was performed using Trans^®^ Script One-Step gDNA Removal and cDNA Synthesis SuperMix (TransGen Biotech, Beijing, China) per the manufacturer’s recommendations. qPCRs were carried out using a 2 × RealStar Green Fast Mixture (GeneStar, Shanghai, China) on an ABI 7500 real-time Detection System (Applied Biosystems, Foster City, CA, USA). The housekeeping gene actin-related protein 4A gene was used as an internal control. The reaction was carried out as follows: 94 °C for 30 s, followed by 40 cycles of 5 s at 94 °C and 34 s at 60 °C. The relative expression levels of the genes were determined with the comparative 2^−ΔΔCt^ method. The primer sequences used in this study are shown in Table S1.

## Figures and Tables

**Table 1:** Properties of the predicted GRF proteins in *M. truncatula* and *M. sativa*.

Gene Name	TIGR Locus	Start Site	End Site	Homologous Gene	PI	MW (kDa)	Protein Length	Subcellular Localization
*MtGRF1*	MtrunA17Chr1g0152191	4965222	4969855	MsG0180000350.01.T01	7.07	70.74	654	Nuclear
*MtGRF2*	MtrunA17Chr2g0300511	18298975	18301839	MsG0280008385.01.T01	8.75	40.80	357	Nuclear
*MtGRF3*	MtrunA17Chr3g0127641	45643490	45646030	MsG0380016593.01.T01	7.8	58.33	540	Extracellular
*MtGRF4*	MtrunA17Chr4g0021261	18408853	18413110	MsG0480020006.01.T01	7.72	37.22	338	Nuclear
*MtGRF5*	MtrunA17Chr4g0070591	60177320	60179812	MsG0880047345.01.T01	7.29	42.01	369	Nuclear
*MtGRF6*	MtrunA17Chr5g0409471	11229054	11232562	MsG0580025368.01.T01	9.03	36.40	325	Extracellular
*MtGRF7*	MtrunA17Chr7g0265931	49112735	49115556	MsG0780041090.01.T01	8.8	42.42	385	Nuclear
*MtGRF8*	MtrunA17Chr8g0343881	7194250	7196469	MsG0880047345.01.T01	8.39	39.89	349	Nuclear
*MsGRF1*	MsG0180000350.01.T01	4753503	4757720	MtrunA17Chr1g0152191	6.28	56.20	516	Nuclear
*MsGRF2*	MsG0280008385.01.T01	30489028	30491429	MtrunA17Chr2g0300511	8.43	41.97	369	Nuclear
*MsGRF3*	MsG0380016593.01.T01	85488062	85491005	MtrunA17Chr3g0127641	7.32	55.45	513	Extracellular
*MsGRF4*	MsG0380016639.01.T01	85977548	85980455	MtrunA17Chr3g0127641	7.81	50.86	463	Extracellular
*MsGRF5*	MsG0480020006.01.T01	31814795	31818355	MtrunA17Chr4g0021261	9.58	34.29	305	Nuclear
*MsGRF6*	MsG0580025368.01.T01	17548590	17551843	MtrunA17Chr5g0409471	8.97	37.23	332	Extracellular
*MsGRF7*	MsG0780041090.01.T01	86436809	86436865	MtrunA17Chr7g0265931	10.25	25.36	224	Nuclear
*MsGRF8*	MsG0880047345.01.T01	85029014	85030631	MtrunA17Chr4g0070591	7.29	42.14	370	Nuclear

## Data Availability

All data in the present study are available in the public database as referred in the Material and Method part.

## Supplementary Materials

The following supporting information can be downloaded at: https://www.mdpi.com/article/10.3390/ijms23136905/s1.
